# Precise colocalization of interacting structural and pigmentary elements generates extensive color pattern variation in *Phelsuma* lizards

**DOI:** 10.1186/1741-7007-11-105

**Published:** 2013-10-07

**Authors:** Suzanne V Saenko, Jérémie Teyssier, Dirk van der Marel, Michel C Milinkovitch

**Affiliations:** 1 Laboratory of Artificial and Natural Evolution (LANE), Department of Genetics and Evolution, University of Geneva, Sciences III, 30, Quai Ernest-Ansermet, 1211 Genève 4, Switzerland; 2 Department of Condense Matter Physics, University of Geneva, Geneva, Switzerland

**Keywords:** Physics of biology, Structural colors, Pigmentary colors, Color patterns, Iridophores, Chromatophores, Reptiles, Lizards, *Phelsuma*

## Abstract

**Background:**

Color traits in animals play crucial roles in thermoregulation, photoprotection, camouflage, and visual communication, and are amenable to objective quantification and modeling. However, the extensive variation in non-melanic pigments and structural colors in squamate reptiles has been largely disregarded. Here, we used an integrated approach to investigate the morphological basis and physical mechanisms generating variation in color traits in tropical day geckos of the genus *Phelsuma*.

**Results:**

Combining histology, optics, mass spectrometry, and UV and Raman spectroscopy, we found that the extensive variation in color patterns within and among *Phelsuma* species is generated by complex interactions between, on the one hand, chromatophores containing yellow/red pteridine pigments and, on the other hand, iridophores producing structural color by constructive interference of light with guanine nanocrystals. More specifically, we show that 1) the hue of the vivid dorsolateral skin is modulated both by variation in geometry of structural, highly ordered narrowband reflectors, and by the presence of yellow pigments, and 2) that the reflectivity of the white belly and of dorsolateral pigmentary red marks, is increased by underlying structural disorganized broadband reflectors. Most importantly, these interactions require precise colocalization of yellow and red chromatophores with different types of iridophores, characterized by ordered and disordered nanocrystals, respectively. We validated these results through numerical simulations combining pigmentary components with a multilayer interferential optical model. Finally, we show that melanophores form dark lateral patterns but do not significantly contribute to variation in blue/green or red coloration, and that changes in the pH or redox state of pigments provide yet another source of color variation in squamates.

**Conclusions:**

Precisely colocalized interacting pigmentary and structural elements generate extensive variation in lizard color patterns. Our results indicate the need to identify the developmental mechanisms responsible for the control of the size, shape, and orientation of nanocrystals, and the superposition of specific chromatophore types. This study opens up new perspectives on *Phelsuma* lizards as models in evolutionary developmental biology.

## Background

Vertebrate skin coloration provides a promising model system for exploring the link between genotypes and phenotypes in an ecological and phylogenetic framework. Indeed, color traits play crucial roles in thermoregulation, photoprotection, camouflage, and visual communication [1-4], and can vary extensively among and within species and populations. Moreover, colors and color patterns are amenable to objective quantification and modeling, providing an opportunity for integrated analyses of phenotypic variation. In particular, non-mammalian vertebrates, for example, squamates (lizards and snakes), exhibit a broad range of pigmentary and structural colors, generated by different types of chromatophores. In addition to melanophores, which produce black/brown melanins, squamates develop xanthophores and erythrophores, containing yellow and red pigments, respectively. These pigments are typically either pteridines, which are synthesized *in situ* from guanosine triphosphate, or carotenoids, which are metabolized from food in the liver and transported to skin via the circulatory system [5,6]. Squamates additionally possess iridophores, which do not contain any pigment but generate structural coloration through interference of light waves with transparent guanine nanocrystals [7-9]. The spatial arrangement of all these cell types generates a broad range of colors and color patterns.

Despite such a high potential for complexity and diversity, lizards and snakes remain relatively under-represented in evolutionary developmental studies in general [10], and in analyses of color-pattern evolution in particular (for example, in comparison with other vertebrates or insects) [3,4,11,12]. Although chromatophores have been described in some squamates [8,9,13-16], and their melanin pathway has been associated with adaptive color variation [17,18], few data are available on the mechanisms that generate extensive variation in non-melanic pigments and structural colors in this lineage. The presence of such a variety of colors in squamates makes them appropriate models for investigating the essentially unknown genetic, developmental, and physical mechanisms generating a diversity of phenotypes through interactions between different types of chromatophores.

Here, we used an integrated approach that combines histology, Raman and UV spectroscopy, mass spectrometry (MS), optics, and numerical simulations to investigate the morphological basis and the physical mechanisms generating variation in color traits in five representative tropical day gecko species of the genus *Phelsuma*. More specifically, we show that 1) the reflectivity of the white belly, and of the dorsal pigmentary red spots and stripes, is increased by underlying, structurally disorganized broadband reflectors, whereas 2) the hue of the vivid dorsolateral background coloration is generated by structural, highly ordered narrowband reflectors, and is modulated both by the photonic crystal geometry and by a layer of yellow pigments. Most importantly, we show that these interactions require precise colocalization of red and yellow pigment cells with iridophores characterized by different organizations of nanocrystals.

## Results and discussion

**Figure 1:**
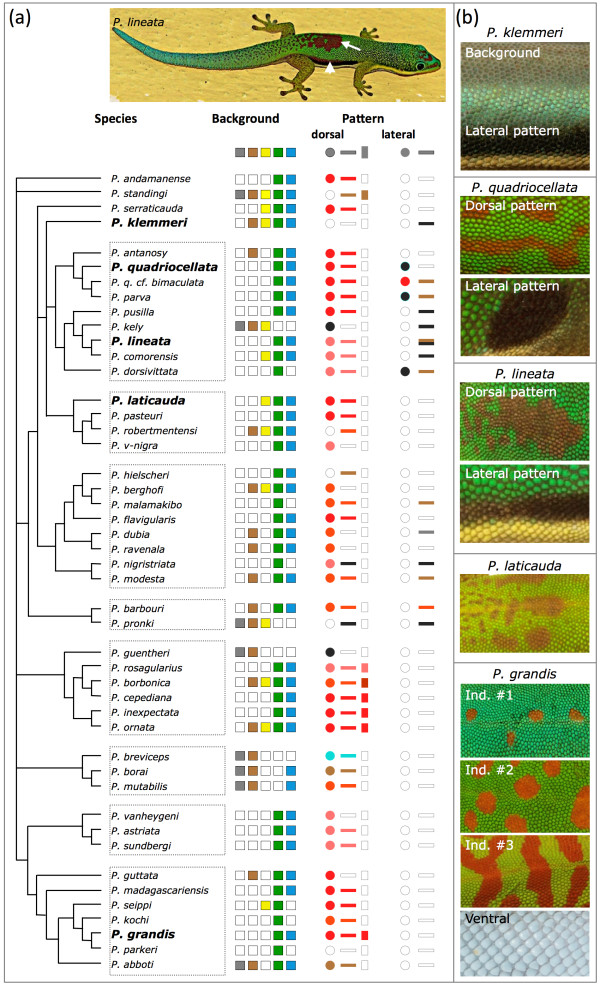
**Color and pattern variation in the genus *Phelsuma. *(a)** Currently recognized species [19,20] with corresponding ranges of background coloration (indicated by filled squares), varying from gray/brown to yellow/green to turquoise-blue, and dorsal and lateral patterns (indicated for illustration on *Phelsuma lineata* with an arrow and an arrowhead, respectively). Empty shapes indicate absence of the corresponding color or pattern. Dorsal patterns vary in both color (typically different shades of red) and shape (spots, stripes, and transverse bars). Lateral patterns appear as spots (sometimes surrounded by a blue ring) or broad stripes. Species indicated in bold were used in this study. **(b)** Skin samples represent variation found across the genus. The skin on the belly (shown here for *Phelsuma grandis*, lowest panel) is off-white in the majority of species.

Tropical day geckos of the genus *Phelsuma* provide excellent models to study the morphological basis of color-pattern variation. Most of the approximately 45 currently recognized species [19,20] are characterized by an off-white ventral and a vivid dorsolateral coloration, with a blue/green background and red (sometimes brownish) spots or stripes on the back (Figure 1a). This vibrant and contrasting color scheme is likely to be involved in mate recognition and/or camouflage [1,2], and is highly variable within and among species. Not only can the background color differ substantially among individuals (as, for example, in *Phelsuma grandis*; Figure 1b, bottom panels), but also the size, shape and intensity of the red dorsal markings. In addition, several species, including *Phelsuma klemmeri*, *Phelsuma quadriocellata* and *Phelsuma lineata*, have lateral black/brown spots or stripes, and a few, such as *P. klemmeri* lack red markings and exhibit a blue/brown background (Figure 1b, top panel).

**Figure 2:**
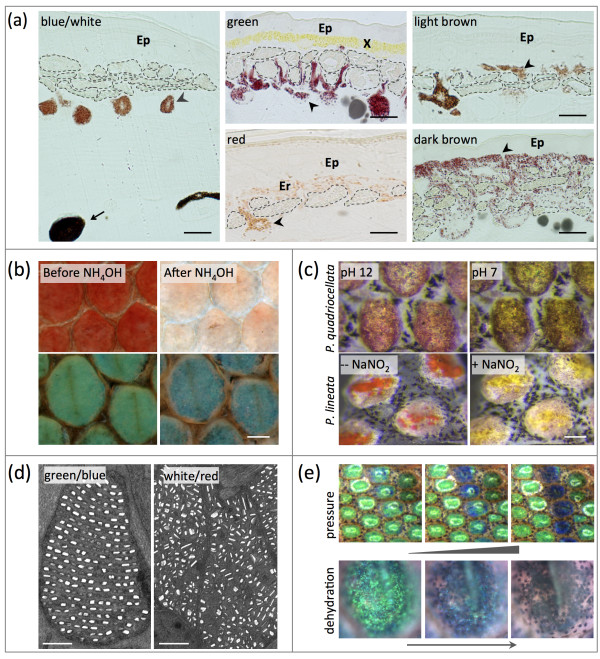
**Pigmentary and structural colorations in *Phelsuma *geckos. (a)** Semi-thin cross-sections of skins of different colors. Two types of melanophores are indicated (arrows and arrowheads, respectively), and iridophores are outlined with dashed lines. Ep, epidermis; X, xanthophores; Er, erythrophores. Bar = 10 μm. **(b)** Pteridin pigments were removed with NH_4_OH (here in *Phelsuma grandis*, individual number 3), revealing the remaining structural color produced by the iridophores. Bar = 0.2 mm. **(c)** Red pigments in dorsal markings of *Phelsuma quadriocellata* and *Phelsuma lineata* can change color when the pH of the Ringer solution is lowered or when an oxidant (NaNO_2_) is added, respectively. Bar = 0.2 mm. **(d)** Representative electron micrographs of iridophores in skin of different colors. Bar = 1 μm. Note the highly disordered guanine crystals in the white and red skin. **(e)** Mechanical pressure and dehydration (here applied to the green skin of *P. grandis*, individual number 2 after removal of the yellow pigment) lead to a blue shift of structural green (for supplementary movies, see Additional file 3; see Additional file 4).

The skin organization in *Phelsuma* geckos (Figure 2a) resembles that of other lizards [9]: chromatophores are absent from the thin epidermis, but are abundant in the thick dermal layer, which contains, from top to bottom, yellow xanthophores or red erythrophores (present in green and red skin respectively), iridophores containing nanocrystals, and dark-brown melanophores. Here, we report on the chemical and optical analyses of pigments and guanine crystals, and provide a multilayer optical model that describes the interactions among different types of pigmentary and structural chromatophores generating a variety of skin colors in *Phelsuma* geckos.

### Melanophores generate dark lateral spots and stripes

The skin of all geckos studied here, regardless of color, contains two types of melanophores (Figure 2a): small light-brown cells that, when associated with iridophores, form ‘dermal chromatophore units’ [21], and large dark-brown cells at the base of the dermis. The former are dendritic cells with processes extending through the layer of iridophores for translocation of melanin granules in response to hormones, resulting in darkening of the skin [9,22]. For example, the melanophore processes cover the iridophores in the dorsal skin of *P. klemmeri*, giving it a light-brown appearance. Moreover, in dark-brown lateral spots and stripes of *P. klemmeri*, *P. quadriocellata*, and *P. lineata*, small melanophores are abundant on top of iridophores, that is, in the layer typically occupied by xanthophores or erythrophores in other parts of the body (Figure 2a). In *P. lineata*, melanophores are found in combination with red erythrophores in the red-brown regions of the lateral stripes. Hence, in addition to their likely involvement in darkening of the skin, the small melanophores form dark lateral spots/stripes in some *Phelsuma* geckos, whereas the larger and darker melanophores might protect the deeper layers from harmful UV radiation [22]. However, the homogeneous distribution of these two types of chromatophores in the skin of any color suggests that neither of them substantially contribute to color variation in *Phelsuma* geckos.

### Pteridins contribute to red and green colors of dorsolateral skin

We could dissolve in NH_4_OH (but not in acetone) both the red and yellow pigments, found respectively in red-skin erythrophores and green-skin xanthophores (Figure 2b). This indicates that both pigments belong to the pteridine class rather than to the carotenoid class [23,24]. We confirmed these findings by Raman spectroscopy and UV spectroscopy/MS (see Additional file 1). The Raman spectra (see Additional file 1: Figure S1) of both yellow xanthophores and red erythrophores in *P. quadriocellata*, *P. lineata*, and *P. laticauda* are similar to that of xanthopterin [25], a known pigment in squamates [15]. Conversely, although the Raman spectrum of yellow xanthophores in *P. grandis* also indicates xanthopterin, the spectrum obtained for the red erythrophores in this species is significantly different, suggesting the presence of other molecules.

We then performed UV chromatography/MS on pigments extracted from the red and green skin of *P. quadriocellata*, *P. lineata*, and *P. grandis*, and compared their spectra with those of available pteridin standards. We confirmed the presence of xanthopterin in the yellow chromatophores of *P. grandis*, and in both the yellow and red chromatophores of *P. lineata*. We also identified biopterin in the yellow and red chromatophores of *P. quadriocellata* and in the red chromatophores of *P. grandis*, and identified sepiapterin in the yellow chromatophores of *P. grandis*. Finally, we found three unidentified molecules (probably pterins, based on their UV absorption spectra (see Additional file 1: Figure S2)) in all three species. It is, however, unclear which components contribute most substantially to the final pigmentary colors of the skin.

Remarkably, although pigment compositions differ substantially between *P. lineata* and *P. quadriocellata*, the pigment compositions of xanthophores and erythrophores are identical within a species (see Additional file 1: Figure S2a), strongly suggesting that the actual *in vivo* colors of these chromatophores (red or yellow) are determined by some other factors, such as the pH of the cellular environment of the pigment (as is the case for plant anthocyanins [26]) or the redox state of the pigment molecules (as has been shown for ommochromes in dragonflies [27]). Supporting this hypothesis, the red color of dorsal skin changed to yellow when we lowered the pH of, or added an oxidant (NaNO_2_) to, the skin of *P. quadriocellata* or *P. lineata*, respectively (Figure 2c). Even though the exact *in vivo* processes generating variation in the pH or redox states are unknown, these results indicate that these mechanisms provide an additional source of color variation in squamates.

### Ordered multilayer interference reflectors generate structural blue or green

In all regions of the body, irrespective of its color, the skin of *Phelsuma* geckos contains iridophores, with guanine nanocrystals, either well-organized in parallel layers (in the light-brown dorsal skin of *P. klemmeri* and in the green/blue skin of all other species), or highly disorganized (in the white and red skin of all animals examined here) (Figure 2d). Using transmission electron microscopy (TEM) images, we measured the orientation of crystals relative to the skin surface, and computed the full-width half maximum (FWHM) of the Gaussian distribution of crystal orientation, and the ratio between the amplitude *A* of the Gaussian curve and the amplitude *y*_*0*_ of the background generated by randomly oriented crystals (see Methods; see Additional file 2: Figure S3d). The *A/y*_*0*_ ratio represents the relative amount of crystals oriented parallel to the skin surface and varies from zero (when *A* = 0 in a purely random system) to infinity (when *A* is high and *y*_*0*_ is close to zero in a perfectly ordered system). Table 1 shows that blue/green skin is characterized by a narrower angular distribution of nanocrystals and a higher *A/y*_*0*_ ratio than white or red skin. This indicates that the ordered nanocrystals generate an intense narrowband structural blue or green color through a multilayer interference mechanism. This result is consistent with the pigment-removal experiments (Figure 2b) discussed above, revealing the structural blue color of iridophores that are typically covered by the yellow pigmentary color of xanthophores in the green skin of some *P. grandis* individuals. Note that crystal spacing (hence, structural color) varies both among and within individuals (Table 1).

**Table 1:** **Geometric parameters of guanine crystals in *Phelsuma *** **species**

**Species (individual)**	**Green/blue**			**White**			**Red**		
	**Orientation *A/y***_***0 ***_**(FWHM)**^**a**^	**Crystal height, nm**^**b**^	**Crystal spacing, nm**^**b**^	**Orientation *A/y***_***0 ***_**(FWHM)**	**Crystal height, nm**	**Crystal length, nm**	**Orientation *A/y***_***0 ***_**(FWHM)**	**Crystal height, nm**	**Crystal length, nm**
*P. grandis* (1)	15.0 (32°)	80.8 ± 12.2/ 78.5 ± 10.9^c^	96.0 ± 20.8/ 104.7 ± 25.5^c^	2.7 (72°)	84 ± 44	173 ± 84	1.3 (82°)	68 ± 34	128 ± 70
*P. grandis* (2)	9.7 (49°)	81.0 ± 12.9	84.9 ± 22.7	2.0 (61°)	78 ± 39	169 ± 96	1.1 (53°)	67 ± 33	124 ± 82
*P. grandis* (3)	13.0 (47°)	79.4 ± 10.7	99.2 ± 10.6	2.6 (59°)	75 ± 36	171 ± 91	1.7 (46°)	71 ± 37	130 ± 73
*P. laticauda*	13.3 (39°)	82.5 ± 11.4	97.3 ± 12.6	2.2 (60°)	71 ± 41	194 ± 140	2.6 (61°)	75 ± 42	154 ± 87
*P. quadriocellata*	20.3 (40°)	70.0 ± 22.7	37.9 ± 13.8	6.4 (49°)	84 ± 42	209 ± 126	4.8 (67°)	69 ± 41	171 ± 103
*P. klemmeri*	9.6 (32°)	68.0 ± 10.8	93.2 ± 26.3	4.5 (58°)	72 ± 47	265 ± 209	NA^d^	NA^d^	NA^d^
*P. lineata*	6.9 (42°)	74.0 ± 11.1	109.6 ± 21.8	4.8 (50°)	94 ± 57	226 ± 137	2.1 (58°)	68 ± 43	170 ± 107

^a^The ratio *A/y*_*0*_ and the full-width half maximum (FWHM) of the Gaussian curve describe the crystal orientation relative to the skin surface (see Methods).

^b^Mean ± standard deviation are given for crystal height and spacing between layers of well-organized crystals in green and blue skin, and for crystal height and length in white and red skin. Note that the parameters describing crystal orientation differ substantially between blue/green and white/red skin.

^c^The two numbers shown for crystal height and spacing of green/blue skin in *P. grandis* individual 1 correspond to samples taken from the back and the neck (blue and yellowish-green, respectively; see Figure 4b). Note that the spacing between crystal layers is substantially larger in the neck sample. Additional data are provided for blue and green skin samples, including a comparison of the simulated colors to the real colors seen in the skin after pigment removal (see Additional file 2: Table S3).

^d^NA, data not available because of the absence of red skin in *P. klemmeri*.

To further test experimentally if the multilayer is responsible for the structural color component of blue/green skin in *Phelsuma*, we applied mechanical or osmotic pressure to modify the distance between the guanine nanocrystal layers and therefore induce changes in structural coloration [16]. We found that mechanical pressure or dehydration applied to de-pigmented green skin (hence, exhibiting structural green) resulted in a shift to structural dark blue (Figure 2e; see Additional files 3 and 4: supplementary movies). Note that complete dehydration made the layer of iridophores transparent, revealing the underlying melanophores (Figure 2e), because a severe decrease in crystal spacing either obliterates coherent interferences (that is, the entire stack of crystals behaves as a single block of guanine), or causes a shift in the reflectivity peak beyond the optical visible range, that is, below 390 nm.

### Optical simulations of ordered multilayer interference match the reflectivities and colors produced by iridophores

**Figure 3:**
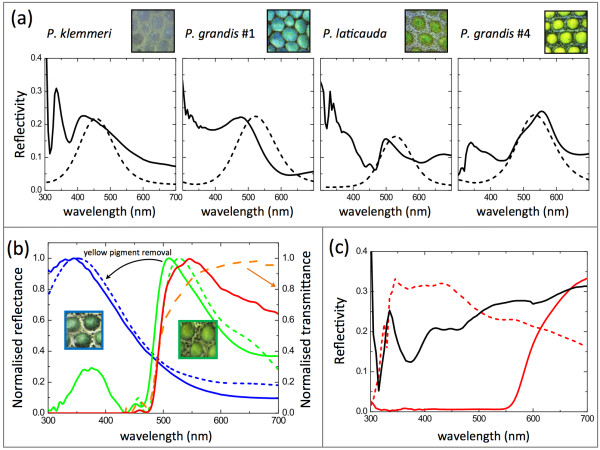
**Experimental and modeled reflectivities of *Phelsuma *skin. (a)** Measured skin reflectivities (solid lines) after removal of pigments (skin colors varied from deep-blue to yellowish-green) compared with modeled reflectivities (dashed lines) based on crystal size and spacing (Table 1). For *Phelsuma grandis* number 4, the crystal geometry parameters were taken from the *P. grandis* number 1 neck sample that exhibited a similar structural color. Note that the UV peaks in the measured reflectivities are probably caused by scattering on melanosomes, on iridophore crystals, or on dermal collagen fibers. **(b)** Normalized reflectivity of green skin before and after yellow pigment removal (green and blue solid lines, respectively). Modeled multilayer responses for a crystal size of 70 nm and a spacing of 30 nm (assuming a standard deviation of 13 nm) are also shown with (dashed green line) and without (dashed blue line) a 3 μm thick pigment layer on top. The direct product of structural blue reflectivity with normalized yellow pigment transmittance (orange dotted line) generates the plain red line, confirming the mechanism of structural color filtering by the top pigment layer. **(c)** Reflectivity measured on *P. grandis* white skin (black line) and on red skin before (red solid line) and after (red dashed line) red pigment removal. Reflectivity intensities of ordered and disordered iridophores are comparable.

We used a multilayer model (Methods; see Additional file 2) to simulate the reflectivity and colors produced by organized crystals using parameters such as crystal height (that is, thickness) and spacing between crystal layers, estimated from TEM images collected for a selection of green and blue skin samples (Table 1; see Additional file 2: Table S3). The measured and modeled reflectivities of four skin samples, covering the range of structural colors seen in the *Phelsuma* individuals studied here, are shown (Figure 3a). Although we did not have TEM data available for the sample of *P. grandis* number 4, its color was similar to that of the *P. grandis* number 1 neck sample. In addition, we compared the RGB (red, green, blue) colors predicted by the model to color pictures of all samples after pigment removal (see Additional file 2: Table S3). Overall, the reflectivities and the RGB colors predicted by our model were similar, but not always identical, to those perceived on the animals. This small discrepancy can be explained by, for instance, changes in cytoplasm osmolarity (and hence, the spacing between crystal layers) during skin preparation for TEM [14-16]. In addition, we found high reflectivity in the UV spectrum (Figure 3a), which might be produced by a series of mechanisms such as coherent scattering on arrays of collagen fibers in the dermis [28] or incoherent scattering [29] on, for example, melanosomes or guanine crystals.

The collaborative interplay between the structural and pigment components is illustrated (Figure 3b); we measured the reflectivity of green skin before and after yellow pigment removal (green and blue solid lines, respectively). The modeled reflectivity for an arbitrarily chosen crystal size (70 nm) and spacing (mean = 30 nm, SD = 13 nm), shown as dashed blue line, closely resembles the measured reflectivity of green skin after pigment removal. When a 3 μm yellow pigment layer is included in the model, the resultant reflectivity (Figure 3b, dashed green line) is a good match to the measured reflectivity of green skin before pigment removal. A similar reflectivity distribution (red line) was obtained as the direct product of the blue line with the measured normalized transmittance of the yellow pigment (orange dashed line). Hence, the green color of the skin is due to the structure-based blue reflection filtered by the yellow-transmitting pigment.

Optical systems based on multilayer interference usually show a spectral shift in reflectivity as the angle of the incident light changes [30]. This angular dependence is determined by the refractive indices of the alternating layers, and the ratio between the optical paths at different angles (that is, path length × the refractive index of the medium) [31,32]. Under very particular combinations of the refractive indices and thicknesses of its two components, a multilayer reflector can exhibit greatly reduced angular dependence [33]. Only one of these conditions is met in *Phelsuma* geckos: the optical paths in low (*n*_*c*_*d*_*c*_) and high (*n*_*g*_*d*_*g*_) index layers are close, making the multilayer a narrowband optimal reflector as a result of the collaborative effects of crystal and spacing interferences. However, the iridescence in *Phelsuma* skin is much weaker than that predicted by the model (see Additional file 5: Figure S5a) because of another reason, namely, the variable orientation of iridophores in the skin reduces or annihilates iridescence by averaging the contributions from different cells (see Additional file 5: Figure S5b).

### Incoherent scattering by disorganized crystals enhances the reflectivity of red dorsal and white ventral skin

Guanine crystals in iridophores of green and blue skin are well-organized, and therefore contribute extensively to the final background color of *Phelsuma* lizards. Remarkably, iridophores are also abundant in both the white ventral skin and red dorsal markings, but these cells are characterized by crystals with mostly random orientations and broad size distribution (Figure 2d; Table 1). This gives rise, as in some insects and fish [34-39], to incoherent scattering. In other words, in contrast to iridophores in blue/green skin, which generate a narrowband distribution of reflected frequencies, iridophores in white and red skin form broadband reflectors with overall reflectivity of similar intensity to that measured on coherently scattering structures (Figure 3c). Hence, these cells with disorganized guanine crystals not only produce the white color of the belly, but also significantly enhance the reflectivity, and therefore the visibility, of the red pigmentary patterns on the back. In the absence of iridophores, the red spots would appear less bright because much of the incident light would be absorbed by the underlying tissues. The mechanisms responsible for the distribution of ordered versus disordered crystals and for this spectacular colocalization of pigment cells with a particular type of iridophores are unknown.

### Variation in skin coloration is explained by both structural and pigmentary components

**Figure 4:**
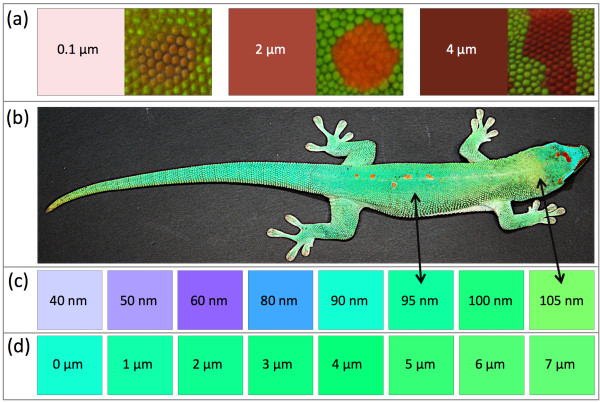
**Colors simulated with the multilayer model. (a)** Red colors simulated with varying thickness (0.1 to 4 μm) of a red pigment layer on top of a white reflector and comparison to red markings of different animals. **(b)***Phelsuma grandis* individual number 1. **(c)** Simulated colors produced by a 7 μm yellow pigment layer on top of a multilayer interference reflector with varying spacing (40 to 105 nm) between layers of crystals 80 nm thick. Double arrows indicate the spacing measured between crystal layers on the dorsal and neck skin of the individual. **(d)** Simulated colors produced with varying thicknesses (0 to 7 μm) of a yellow pigment layer on top of a blue reflector.

Variation in background coloration and dorsolateral patterns in *Phelsuma* geckos (Figure 1) can be generated by changes in pigmentary and structural components. For example, variation in the intensity of the red dorsal patterns can be caused by differences in the density of erythrophores in the top dermal layer and/or in concentrations of red pigment in these cells. The color variation obtained from a simulated red pigment layer of variable thickness (0.1 to 4 μm) on a fully reflecting background was a good match to the variation in intensity of the red markings seen among species or individuals (Figure 4a). Similarly, different densities of xanthophores and/or concentrations of yellow pigment, in combination with structural colors produced by iridophores, can lead to variation from blue to green. For example, the background skin color of *P. grandis* individual number 1 (Figure 4b) varies from a greenish-blue to yellowish-green depending on the area of the body involved. This variation is explained by an increase of about 10% in spacing between guanine crystals (96.0 nm in the back skin versus 104.7 nm in the neck skin; Table 1). The colors of these two different zones on the skin (Figure 4b) are in agreement with the predictions of our optical model (Figure 4c). Note that similar variation in skin color could also be due to modification of the thickness or density of the yellow pigment layer without any change in the structural component (Figure 4d).

## Conclusions

Extensive variation in background coloration, and in dorsal and lateral color patterns, is present within and among species of the genus *Phelsuma* (Figure 1). Our analyses indicate that this variation is generated by a combination of features associated with pigmentary and structural color chromatophores (as previously reported in birds [40,41]). The black lateral spots and stripes found in some species, and the light-brown background coloration of *P. klemmeri*, are due to melanophores occupying the upper layers of the dermis. Blue skin color is solely due to iridophores with well-organized guanine nanocrystals (that is, narrowband multilayer interference reflectors), whereas green skin is produced, depending on the species and the individual, either by structural green or by the interaction of structural blue with yellow pigments (xanthophores). Dorsal marks are formed by red erythrophores, the reflectivity of which is enhanced by iridophores with disorganized crystals (that is, broadband reflectors producing incoherent scattering). In addition, the hue produced by erythrophore and xanthophore pigments is pH-dependent or redox-dependent in some species. Most importantly, we show that the color patterns of *Phelsuma* always require precise colocalization of different sets of interacting pigmentary and structural elements. For example, yellow and red chromatophores are associated with iridophores with ordered and disordered nanocrystals, respectively. This indicates the need to identify the developmental mechanisms responsible for the superposition of specific chromatophore types, opening up new perspectives for *Phelsuma* lizards as model organisms in evolutionary developmental biology.

We also show that the exact combination of parameters producing a given color is difficult to predict without experimental estimation of parameter values (such as*,* through electron-microscopy and spectroscopy analyses). Hence, exploring the genetic and developmental bases of phenotypic variation in ecologically important color traits in squamate reptiles will require integration of the structural and pigmentary color components and their interactions. The fact that red/yellow coloration in *Phelsuma* is based on pteridines, the synthesis of which is controlled by enzymes that have been well studied in model organisms [42], readily suggests candidate genes for this pigmentary aspect of color variation. Furthermore, although guanine crystal formation in iridophores is poorly understood, and the genetic determinants of variation in size, shape, and orientation of nanocrystals are virtually unknown, several enzymes associated with aberrant iridophore phenotypes [43,44] are candidates for variation in structural coloration in *Phelsuma*.

## Methods

Maintenance of, and experiments on, animals were approved by the Geneva Canton ethical regulation authority (authorization 1008/3421/1R) and performed in accordance with Swiss law.

### Animals and phylogenetic mapping

Adult *P. klemmeri* (n = 1), *P. quadriocellata* (n = 1), *P. lineata* (n = 2), *P. laticauda* (n = 1), and *P. grandis* (n = 5) were obtained from the pet market or bred in our laboratory. On the phylogenetic tree of the genus *Phelsuma*[19,20], we mapped (Figure 1a) the presence or absence of the following phenotypic characters (based on previous reports and our own observations [20,45-50]): 1) dorsolateral background coloration; 2) red/brown dorsal pattern; and 3) black/brown lateral pattern. Most *Phelsuma* geckos exhibit a vivid dorsolateral coloration, with a background typically of one or more colors (for example, bright green, yellow, or turquoise-blue, but sometimes dull gray or brown). Almost all species have distinct dorsal marks consisting of red to brown spots and stripes of various shapes. In addition, some species have black/brown lateral stripes or spots.

### Skin histology and TEM

Skin samples were placed in Ringer’s solution for microscopy observation. Cross-sections of 14 to 16 μm were prepared from skin embedded in optimum cutting temperature compound on a Leica (Wetzlar, Germany) CM1850 cryostat. For TEM, skin pieces of approximately 1 cm^2^ in size were fixed overnight at 4°C in 2% glutaraldehyde and 4% paraformaldehyde in 0.05 mol/L sodium cacodylate buffer (pH 7.4), rinsed with 0.1 mol/L cold sodium cacodylate, and fixed for 1.5 hours on ice in the same buffer with the addition of 1% osmium tetraoxide. Samples were then rinsed in water, stained with 1% uranyl acetate for 2 hours, gradually dehydrated in ethanol, rinsed three times in propylene oxide, incubated overnight in 1:1 propylene oxide/resin, and finally embedded in epoxy resin (Epon). Semi-thin (1 to 2 μm) and ultra-thin (80 to 90 nm) cross-sections were cut with a diamond knife on a Leica UCT microtome. Semi-thin sections were examined under a light microscope. Ultra-thin sections were placed on formavar-coated grids, and viewed with a Tecnai™ G2 Sphera (FEI) TEM at 120 kV to visualize intact guanine crystals, because post-staining inevitably results in loss of crystals [13]. The grids were then post-stained with uranyl acetate and lead citrate, and viewed with the TEM again (in this case, spaces that were occupied by crystals appear white).

### Optical modeling of skin reflectivity and color

For each individual, 10 to 20 TEM images of stained sections of iridophores of white, red, and green skin were collected at low (×1,500) magnification, and 20 to 30 images of unstained sections were collected at high (×25,000) magnification for green/blue skin. To analyze the size and orientation of crystals relative to skin surface, white ‘holes’ (corresponding to guanine crystals dissolved by uranyl acetate/lead citrate during post-staining) on low magnification images were fitted with ellipses (in JMicroVision [51]), whose geometric parameters (length, height, and orientation of major axis) were computed subsequently.

The Gaussian distribution of crystal orientation was calculated as:

$$y\left(\theta \right)=y_{0}+Ae^{-\frac{{\left(\theta -\theta_{c}\right)}^{2}}{2w^{2}}},$$

where *y*_*o*_ is the intersection of the curve with the y-axis (that is, the background level of randomly oriented crystals); *A* is the amplitude of the Gaussian curve; *θ* is the angle of the smallest axis of the ellipse relative to the normal to the skin surface; *θ*_*c*_ is the central value of the distribution, and is equal to 0; and *w*, the standard deviation, is related to the FWHM of the Gaussian curve, defined as

$$2\sqrt{2\times \ln\;\left(2\right)}\times w.$$

The *A/y*_*0*_ ratio varies from zero (when *A* = 0 in a purely random system) to infinity (when *A* is high and y_0_ is close to zero in a perfectly ordered system) as schematically illustrated (see Additional file 2: Figure S3d).

For optical modeling in blue/green skin, the thickness of crystals and the spacing between well-aligned crystal layers were measured on images of unstained sections taken at ×25,000 magnification (Table 1; see Additional file 2: Table S3). These values were used in a multilayer model (see Additional file 2) that simulates the reflectivity and color produced by alternating layers of guanine crystals and cytoplasm, each with a different thickness and refractive index [14,52,53].

To account for the contribution of pigments to skin reflectivity and hue, a top layer of pigment with variable thickness and wavelength-dependent absorption was included in the model. The optical transmission of the pigments measured on skin cryosections was fitted to a Drude-Lorentz model and included in the multilayer (see Additional file 2). As the intensity of light reflected coherently by organized photonic structures is much more intense (reflectivity can be close to 1) than that reflected by incoherent scattering, the latter was not taken into account.

### Pigment chemical analysis

To determine pigment composition in xanthophores and erythrophores, skin samples were treated with 100% acetone and 30% NH_4_OH, known to dissolve carotenoids and pteridins, respectively [23,24]. MS was performed on pteridin standards (neopterin, isoxanthopterin, xanthopterin, pterin, 6-biopterin and sepiapterin) and on extracted pigments by UHPLC/DAD/ESI-QTOF (Agilent Technologies 1290 Infinity, ESI-QTOF model G6530A). A Poroshell C8 analytical column (100 × 3.0 mm internal diameter; particle size 2.7 μm) was used for the separation. The column oven temperature was set at 40°C. The binary mobile phase consisted of 0.4% acetic acid in water (solvent A) and acetonitrile (solvent B), and the flow rate was set to 0.5 ml/min. UV/visible spectra were recorded between 190 to 600 nm, and the injection volume was 1 μl. Mass spectra were acquired in positive and negative mode using electrospray ionization with nitrogen as the nebulizing gas, and recorded for the range of *m/z* 50 to 1700. Drying gas flow was 10 l/min, with a fragmentation voltage of 120 V, drying gas temperature of 300°C, nebulizer pressure of 40 psi, and capillary voltage of 3500 V. Compounds were identified with MassHunter Qualitative Analysis Software (Agilent Technologies) by analysis of their UV and high-resolution MS spectra (see Additional file 1).

Raman spectroscopy was performed on cryosections of red and green/yellow skin. This non-invasive technique [54] determines the chemical structure of a target molecule by measuring the energy shift of an incident laser light after its interaction with the molecule. The bespoke micro-Raman system was composed of a half-meter focal length spectrometer coupled to a nitrogen-cooled Princeton CCD detector. The excitation source was an argon laser with a wavelength of 514.5 nm. The full collection of Raman spectra on different pigments is shown (see Additional file 1).

### Optical measurements

For extended UV reflectivity measurements (300 to 800 nm), a probe (QR400-7-VIS/BX; Ocean Optics) was connected to the scanning Xenon source of a spectroscopic Woollam ellipsometer with silicon diode as detector. Lock-in detection was used for ambient light noise reduction. For monitoring color change in real time (see Additional files 3 and 4, movies) and for imaging of the skin samples (see Additional file 2: Table S3), a Thorlab color CCD camera was mounted on a Leica M125 stereo microscope with a high field depth. With CCD, a systematic underestimate of RGB numbers of about 5% (that is*,* darker colors) was seen, but the color balance was rendered correctly. During the mechanical pressure and dehydration experiments, skin color was measured *in situ*, and compared with simulations in which the distance between the crystals was the only adjusted parameter.

## Competing interests

The authors declare that they have no competing interests.

## Authors’ contributions

MCM conceived and supervised the whole study; SVS performed phylogenetic mapping, pigment extraction, and TEM.; SVS and JT performed the color analyses; JT and DvdM performed the numerical simulations; and MCM, SVS, and JT wrote the manuscript. All authors commented on the manuscript and approved the final version.

## Supplementary Material

Additional file 1 **Pigment analyses.** Results of Raman and UV/mass spectroscopy on pigments. Click here for file

Additional file 2 **Optical model.** Description of the optical model used to simulate skin colors. Click here for file

Additional file 3 **Movie of mechanical pressure in *Phelsuma *species.** Real-time movie showing how pressure manually applied with tweezers locally modified the structural color, and how the relaxation process drove the skin back to its original color. Note that the yellow pigments had been removed before the experiment. Click here for file

Additional file 4 **Movie of osmotic pressure in *Phelsuma *species.** Accelerated movie (×16) showing the effect of dehydration (in air) and rehydration on the structural color generated by iridophores. Note that the yellow pigments had been removed before the experiment. Click here for file

Additional file 5 **Angular dependence.** Angular dependence of the multilayer reflectivity in green skin of *Phelsuma grandis*. Click here for file
