# Supplementary material for: Precise colocalization of interacting structural and pigmentary elements generates extensive color pattern variation in Phelsuma lizards
Source: BMC Biol. 2013 Oct 7;11:105. doi: 10.1186/1741-7007-11-105 (PMC4021644; doi:10.1186/1741-7007-11-105)
Supplement: Additional file 1 — Pigment analyses. Results of Raman and UV/mass spectroscopy on pigments. [file 1741-7007-11-105-S1.pdf]

Precise colocalization of interacting structural and pigmentary elements generates extensive color pattern variation in *Phelsuma* lizards

Suzanne V. Saenko, Jérémie Teyssier, Dirk van der Marel, & Michel C. Milinkovitch

Additional file 1:
Results of Raman and UV/mass spectroscopy on pigments.

We performed Raman spectroscopy on pigment cells in cryosections of red and green skin (Figure S1). Because Raman spectroscopy generates a single spectrum for all molecules present in the sample, this type of analysis gives a general idea of what kind of pigments (*i.e.* melanins, carotenoids, or pterins) is present, but does not allow to precisely identify the pigments that contribute to skin color. Here, the similarity of Raman spectra of the majority of samples to that of xanthopterin [1] supports the presence of pterins in red/green skin of *Phelsuma* lizards. To further identify pigment composition of these cells, we then performed UV chromatography / mass spectrometry analyses on red and yellow pigments of *P. grandis*, *P. lineata* and *P. quadriocellata*, with, as a reference, available pterin standards measured in the same experimental conditions. The main properties of the identified molecules are shown in Table S1.

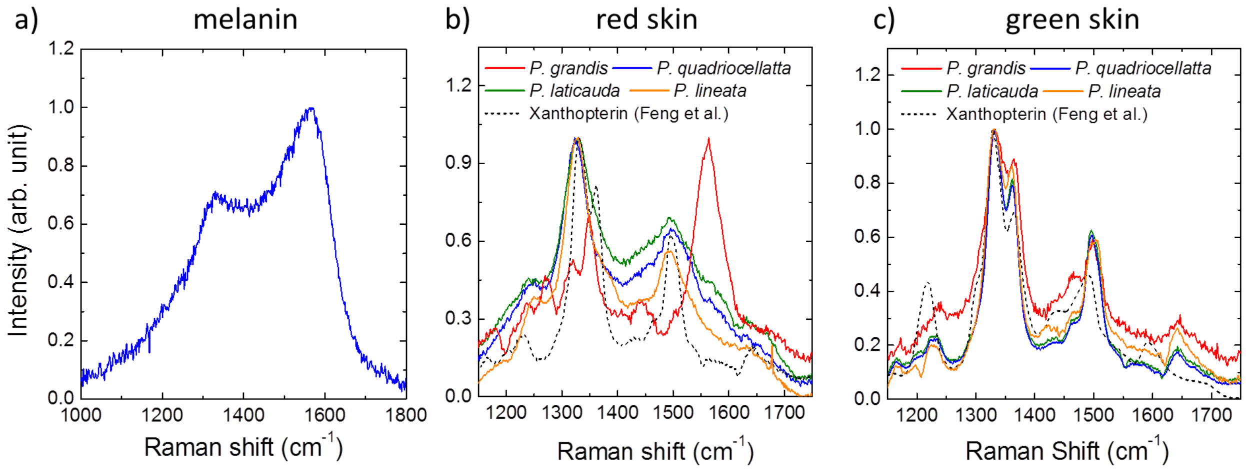

Figure S1. (a) The Raman spectrum of melanin found in both types of melanophores in *P. grandis*. **(b)** The spectra of pigments found in erythrophores of red skin in *P. laticauda, P. lineata* and *P. quadriocellata* resemble that of xanthopterin reported in Feng *et al.* [1], except for the absence of a 1370 cm^{-1} peak, which could be due to a different conformation or environment of the molecule. In contrast, the energy position of peaks in the Raman spectrum of the red skin in *P. grandis* is shifted to the right, suggesting the presence of other molecules contributing to Raman. **(c)** The spectra of pigments found in xanthophores of green skin match well with that of xanthopterin reported in Feng *et al.* [1].

Table S1. Column retention times, for standards (in bold) as well as red and yellow pigments, detected by UV (RT UV) chromatography or mass spectrometry (RT Mass). The name of pterins (if identified), the position of main peaks in the UV spectrum (λ max) and the mass (MS) are indicated for each component. Numbers 1-9 are used for peak identification in Figure S2.

number	RT UV (min)	RT Mass (min)	name	λ max (nm)	MS
1	1.275	1.345	**neopterin**	198 212 274 340	254.0900
2	1.489	1.566	not identified	222, 298, 338	196.0477
3	1.632	1.707	not identified	198, 214, 274, 384	180.0526
4	1.879	1.948	**isoxanthopterin**	198, 210, 286, 340	180.0530
5	2.008	2.073	**xanthopterin**	212, 286, 340	180.0523
6	2.165	2.238	**pterin**	196, 216, 230, 270, 332	164.0571
7	2.269	2.354	**6-biopterin**	196, 214, 230, 272, 336	238.0948
8	2.384	2.453	**sepiapterin**	196, 218, 234, 274, 338	238.0950
9	2.534	2.608	not identified	196, 216,236, 290, 344	208.0467

The Diode Array detected (DAD) plots (Figure S2a) allow identification of some molecules present in pigment extracts from yellow and red skin of *Phelsuma* lizards. For each peak in the DAD plots, we compared the mass and the UV-absorption spectrum of the corresponding compound to those of the pterin standards (Figure S2b). The UV spectra of molecules 2, 3 and 9 (Figure S2c) resemble those of the pterin standards (*i.e.* a high peak around 200 nm followed by one or two lower peaks at about 270-400 nm [2]), suggesting that these could be pterins as well. For some DAD plot peaks, the mass is in agreement with the standard, but the shape of the UV absorption is either weak or deviating from that of the standard. Such ambiguous peaks are indicated in grey in Figure S2a (*e.g.*, the UV spectrum of compound 9 in *P. lineata* has a high peak around 200 nm, but does not have additional peaks at 270-400 nm). The absence of DAD plot peak 5 in *P. quadriocellata* suggests the absence of xanthopterin and seems therefore to contradict Raman results (Figure S1). However, peak 3 with the same molecular mass (Table S1) likely corresponds to xanthopterin in a different configuration.

Although the DAD absorption plots are significantly different between *P. lineata* and *P. quadriocellata*, the plots for red and yellow pigments are remarkably similar within each of these two species, suggesting that the red and yellow chromatophores have identical pigment compositions and that their actual *in vivo* colours are determined by some other factors, *e.g.* the pH of pigment cellular environment or the redox state of the pigment molecules (see Figure 2c in main text).

Notably, the UV absorption peaks measured here for all pterins are in the range of 200-400 nm. Absorption outside of the visible spectrum seems contradictory for red-yellow pigments but can be explained by: *(i)* very low concentration of the molecules in solution (*e.g.*, absorption spectra of xanthopterin vary with concentration [3]), *(ii)* differences in the chemical environment of the molecules, *e.g.* pH [4, 5]. Here, analyses have been performed in a mixture of 0.4% acetic acid (in water) and CH_3CN (pH = 4-5). This is very likely to cause protonation of nitrogen sites and thus modification of charge transfer resulting in a blue shift of absorption wavelengths.

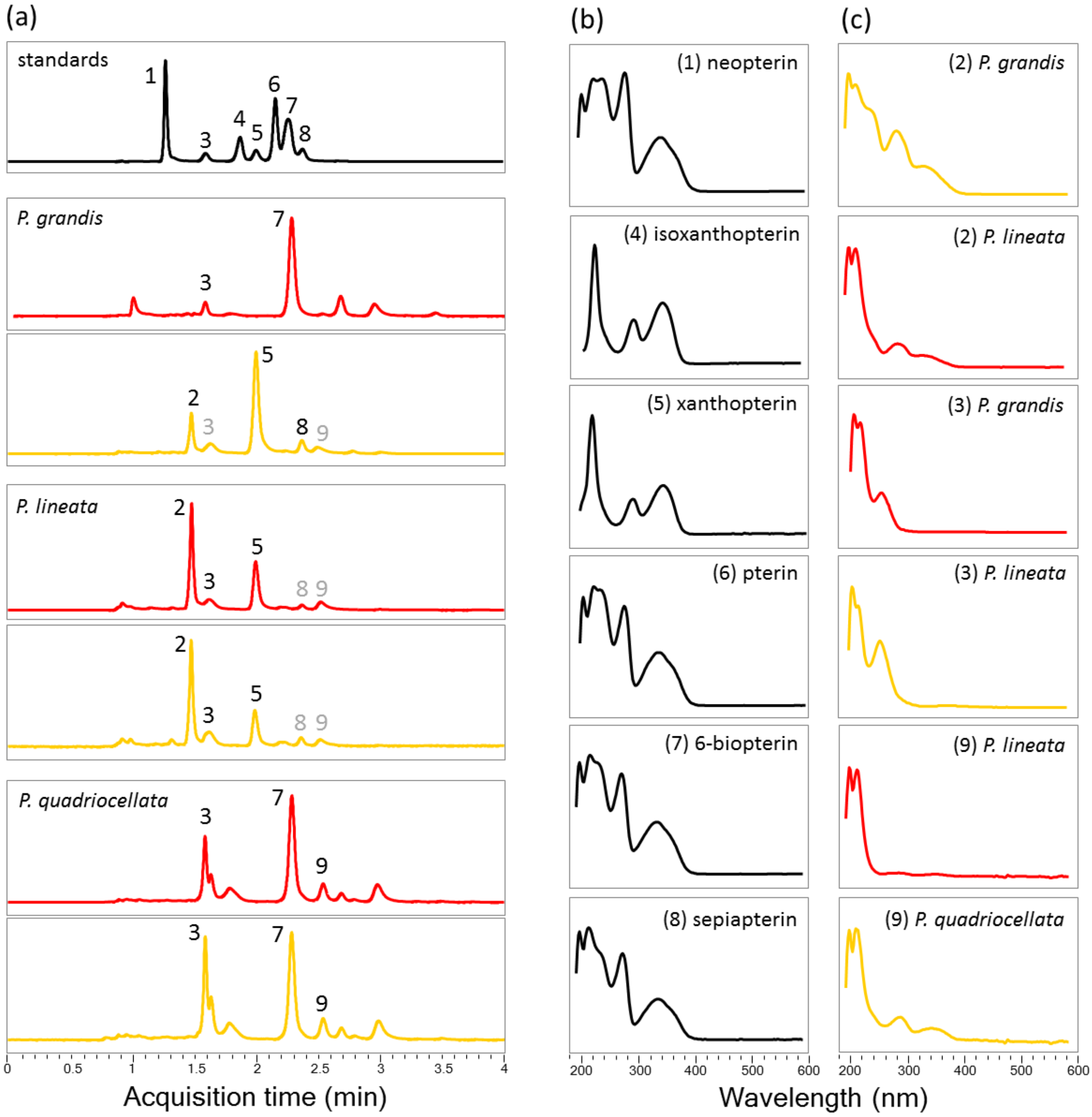

Figure S2. (a) DAD plots showing retention times at 320 nm for the mixture of pterins in a standard and in extracts from *Phelsuma* red and green skin (*i.e.*, red and yellow plots, respectively). The main peaks are numbered according to Table S1 (ambiguous peaks are indicated in grey). (b) UV absorption spectra (arbitrary linear unit scale) of the standards. (c) UV absorption spectra of unidentified compounds found in pigment extracts from red or green skin of different animals (indicated by red and yellow plots, respectively). Numbers correspond to components in Table S1. The UV spectra of compounds 2, 3 and 9 are similar to those of other pterins, hence the corresponding molecules are likely to be pterins as well.

References:

1. Feng Z, Liang C, Li M, Chen J, Li C: **Surface-enhanced Raman scattering of xanthopterin adsorbed on colloidal silver.** *J Raman Spectr* 2001, **32**:1004–1007.
2. Thomas AH, Lorente C, Roitman K, Morales MM, Dántola ML: **Photosensitization of bovine serum albumin by pterin: A mechanistic study.** *J Photochem Photobiol B: Biol* 2013, **120**:52-58.
3. Wijnen B, Leertouwer HL, Stavenga DG: **Colors and pterin pigmentation of pierid butterfly wings.** *J Insect Physiol* 2007, **53**:1206–1217.
4. Cabrerizo FM, Dántola ML, Thomas AH, Lorente C, Braun AM, Oliveros E, Capparelli AL: **Photooxidation of pterin in aqueous solutions: biological and biomedical implications.** *Chem Biodivers* 2004, **1**:1800-1811.
5. Melber C, Schmidt GH: **Identification of fluorescent compounds in certain species of Dysdercus and some of their mutants (Heteroptera: Pyrrhocoridae).** *Comp Biochem Physiol* 1992, **101**:115-133.
