# Supplementary material for: Precise colocalization of interacting structural and pigmentary elements generates extensive color pattern variation in Phelsuma lizards
Source: BMC Biol. 2013 Oct 7;11:105. doi: 10.1186/1741-7007-11-105 (PMC4021644; doi:10.1186/1741-7007-11-105)
Supplement: Additional file 2 — Optical model. Description of the optical model used to simulate skin colors. [file 1741-7007-11-105-S2.pdf]

Precise colocalization of interacting structural and pigmentary elements generates extensive color pattern variation in *Phelsuma* lizards

Suzanne V. Saenko, Jérémie Teyssier, Dirk van der Marel, & Michel C. Milinkovitch

Additional file 2: Description of the optical model

The multilayer is composed of guanine crystals and cytoplasm as sketched in the Figures S3a,b. Although analytical expressions for the reflectivity and transmission can be written for a single crystal as for a multilayer, the reflectivity is numerically evaluated using transfer matrix methods [1] available in the optical spectroscopy package Reffit [2].

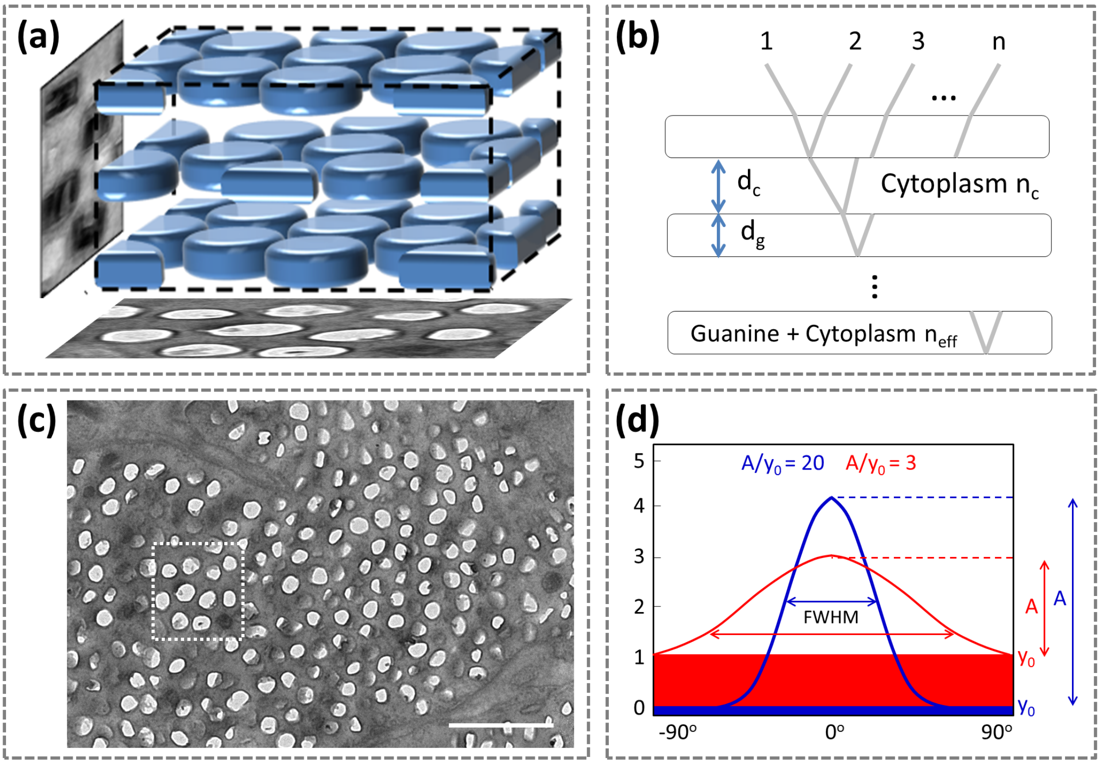

Figure S3. (a) Schematic 3D view of parallel layers of guanine crystals in iridophores, with TEM images of cross- and longitudinal sections projected on the left and bottom, respectively. **(b)** Schematic representation of the multilayer interference optical model. **(c)** TEM image of the longitudinal section (dashed frame corresponds to the image on the bottom of panel **a**), showing absence of crystal order (bar = 1 micron). **(d)** Schematic representation of Gaussian curves describing crystal orientation relative to skin surface in white/red (red line) and blue/green (blue line) skin samples, as well as the parameters A, y_0 and FWHM. The areas filled with red and blue represent the 'background' levels of crystals oriented at all possible angles.

Given that the guanine layer is made of tablet-shaped crystals (refractive index n_g ~1.83) dispersed in cytoplasm (refractive index n_c ~1.34), and that the distance among crystals in a single layer is much smaller than the wavelength of light passing through, each layer of crystals can be treated as an effective continuous medium, as described in Maxwell-Garnett approximation [3]

(implemented in Reffit). We therefore calculated the effective dielectric function of crystal layers as:

$$\varepsilon_{eff} = \varepsilon_c \frac{\varepsilon_g(1+2\delta)-\varepsilon_c(2\delta-2)}{\varepsilon_c(2+\delta)+\varepsilon_g(1-\delta)} = n_{eff}^2,$$

where $\varepsilon_g = n_g^2$ and $\varepsilon_c = n_c^2$ are real part of the dielectric functions of guanine and cytoplasm respectively, and δ is the volume fraction occupied by guanine crystals. The latter was measured on TEM images of longitudinal sections (see Figure S3c) as ~60%, which is in agreement with previous reports [4]. Therefore, the effective refractive index $n_{eff} = \sqrt{\varepsilon_{eff}} = 1.62$ was used in the model instead of the refractive index of guanine reported in previous studies.

Solving the optical response of a multilayer can be done analytically for each wavelength considering the intrinsic optical properties of individual constituents. However, the relatively smooth frequency dependence in the visible range of dielectric functions of the different materials stacked in the multilayer can be very well captured by the Drude Lorentz (D-L) formalism, in which the frequency-dependent complex dielectric function $\tilde{\epsilon}(\omega)$ (*i.e.* complex refractive index $\tilde{\epsilon}(\omega) = \tilde{n}(\omega)^2$) is described as a sum of Lorentz harmonic (damped) oscillators:

$$\tilde{\epsilon}(\omega) = \epsilon_\infty + \sum_i \frac{\omega_{pi}}{\omega_{oi} + \omega - i\gamma_i\omega}$$

Here, ϵ_∞ is the so-called "high-frequency dielectric constant", which represents the contribution of all oscillators at very high frequencies (compared to the frequency range under consideration). The parameters ω, ω_{pi}, ω_{oi} and γ_i are the frequencies of the incoming light, the "plasma" frequency, the transverse frequency (eigen-frequency) and the line-width (scattering rate) of the *i*-th Lorentz oscillator, respectively. D-L modeling drastically reduces the number of parameters and thus allows real-time computation. Moreover, it constitutes a unified basis for the different inputs to the multilayer. Indeed, D-L parameters were obtained by fitting guanine and cytoplasm refractive indices and absorptions taken from the literature (see references in the main text). The scattering parameters γ_i were fixed to a large value so that refractive indices are constant over the entire frequency range. In the case of red and yellow pigments, D-L parameters were obtained by fitting transmission measurements on thick (20 microns) skin cryosections (Figure S4). The sections were performed on a Leica DM 2500 P microscope with an Ocean Optics HR2000+ spectrometer (spectral range 440-835 nm, resolution 0.5 nm) connected to the ocular through an optical fiber. D-L parameters of the different materials (Table S2), as well as geometric parameters (*i.e.*, crystal size and spacing) measured on TEM images of blue/green skin sections (Table S3) were used as input in Reffit to compute optical response.

The influence of the standard deviation of multilayer geometric parameters on reflectivity was calculated in 2 steps. First, the crystal size was set to the average value and the reflectivity is calculated for 100 different spacing values in an interval spreading over four times the standard deviations and centered on the average values. Then, the reflectance was calculated by summing the different contribution with a Gaussian weight factor. The same operation is repeated fixing the spacing to the average value and varying crystal size. Total reflectivity is the average between fixed crystal size and fixed crystal spacing reflectivities. Color was computed by convoluting the reflectivity with standard x,y,z spectral functions.

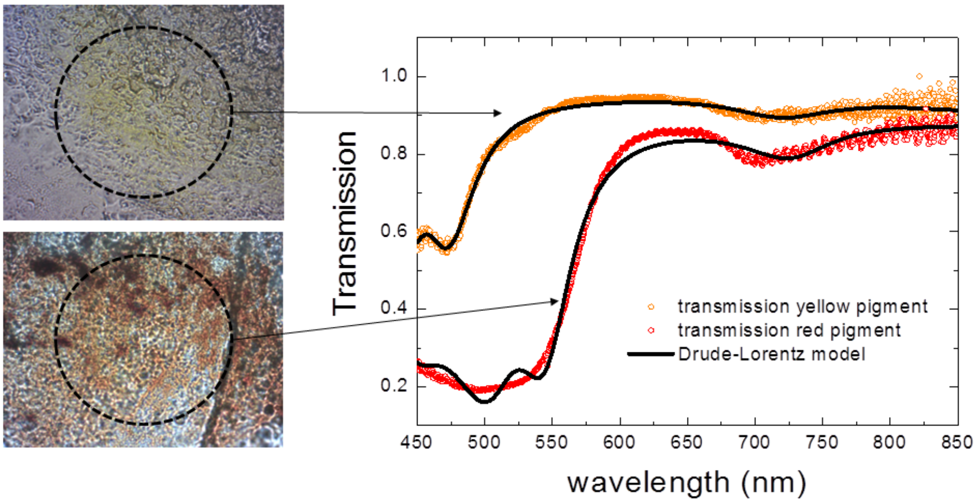

Figure S4. Transmission measurements on yellow and red pigments (symbols) and Drude-Lorentz modeling (black lines) on *P. grandis*. Left pictures show the areas of skin cryosections on which measurements were made.

Table S2. Drude-Lorentz model parameters for the different materials used as input to model skin colour.

Red pigment		ε_∞	0.242
Oscillator #	ω_0 (cm^{-1})	ω_p(cm^{-1})	γ (cm^{-1})
1	13710	967	1424
2	18344	3431	1179
3	19778	4137	1709

Yellow pigment		ε_∞	1.79
Oscillator #	ω_0 (cm^{-1})	ω_p(cm^{-1})	γ (cm^{-1})
1	11402	453	2282
2	13889	450	1724
3	21161	1428	1945
4	22414	848	1058

Cytoplasm		ε_∞	1.79
Oscillator #	ω_0 (cm^{-1})	ω_p(cm^{-1})	γ (cm^{-1})
1	0	636	1e5

Guanine		ε_∞	3.35
Oscillator #	ω_0 (cm^{-1})	ω_p(cm^{-1})	γ (cm^{-1})
1	0	5809	1e5

Table S3. Geometric parameters of guanine crystals in blue and green skin. The ratio A/y_0 and the full-width half maximum (FWHM) of the Gaussian curve describe crystal orientation relative to skin surface. Mean and standard deviation (SD) are given for crystal height and spacing between layers of well-organized crystals. The effective colors simulated from these parameters and compared to the real colors observed on the skin after pigment removal. For a more straightforward comparison between the modeled color and the real color, we compare fully saturated images and [RGB] vectors normalized to the highest R, G or B value.

**Individual**	***P. grandis #1***			***P. grandis #2***		***P. grandis #3***		***P. laticauda***		***P. quadriocellata***				***P. klemmeri***		***P. lineata***
**# samples**	**3**			**2**		**2**		**2**		**4**				**2**		**1**
Orientation A/y_0 [FWHM]	15.0 [32°]			9.7 [49°]		13.0 [47°]		13.3 [39°]		20.3 [40°]				9.6 [32°]		6.9 [42°]
# measured	4766			2121		2760		3627		27136				8479		10194
sample #	**1**	**2**	**3**	**1**	**2**	**1**	**2**	**1**	**2**	**1**	**2**	**3**	**4**	**1**	**2**	**1**
Height [SD] in nm	80.7 [11.2]	80.9 [13.1]	78.5 [10.9]	80.6 [12.2]	81.4 [13.5]	80.0 [10.9]	78.8 [10.6]	82.4 [11.8]	82.6 [11.0]	67.4 [15.2]	72.0 [41.1]	70.0 [13.3]	70.8 [10.7]	66.4 [10.9]	69.7 [10.4]	74.0 [11.1]
# measured	146	162	166	142	179	157	164	187	171	190	159	203	161	208	193	51
Spacing [SD] in nm	94.3 [23.9]	97.7 [17.0]	104.7 [25.5]	96.7 [18.7]	75.0 [21.1]	104.6 [17.5]	94.2 [20.2]	97.2 [12.1]	97.4 [13.2]	33.8 [15.1]	34.1 [8.9]	42.6 [15.2]	41.5 [12.1]	90.2 [21.7]	96.6 [30.4]	109.6 [21.8]
# measured	114	113	127	100	119	127	136	156	127	130	120	130	119	165	150	51
Effective colour R	15	8	94	11	95	119	17	39	45	218	223	200	201	107	12	66
Effective colour G	255	255	255	255	142	255	255	255	255	227	222	205	206	115	208	255
Effective colour B	157	132	115	140	255	108	180	124	121	255	255	255	255	255	255	122
Real colour	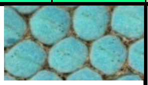		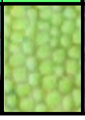	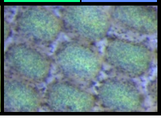		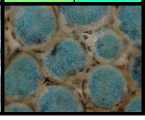		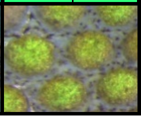		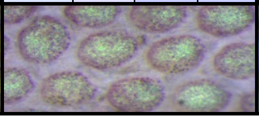				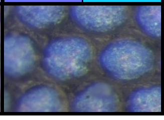		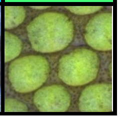

References

1. Harbecke B: **Coherent and incoherent reflection and transmission of multilayer structures.** Appl Phys A 1986, **39**:165-170.
2. Kuzmenko AB: **Kramers–Kronig constrained variational analysis of optical spectra.** *Rev Sci Instrum* 2005, **76**:083108.
3. Maxwell-Garnett JC: **Colours in metal glasses and metal films.** *Philos Trans R Soc London* 1904, **203**:385-420.
4. Rohrlich ST, Porter KR: **Fine structural observations relating to the production of color by the iridophores of a lizard *Anolis carolinensis*.** *J Cell Biol* 1972, **53**:38-52.
