# Supplementary material for: Precise colocalization of interacting structural and pigmentary elements generates extensive color pattern variation in Phelsuma lizards
Source: BMC Biol. 2013 Oct 7;11:105. doi: 10.1186/1741-7007-11-105 (PMC4021644; doi:10.1186/1741-7007-11-105)
Supplement: Additional file 5 — Angular dependence. Angular dependence of the multilayer reflectivity in green skin of Phelsuma grandis. [file 1741-7007-11-105-S5.pdf]

Precise colocalization of interacting structural and pigmentary elements generates extensive color pattern variation in *Phelsuma* lizards

Suzanne V. Saenko, Jérémie Teyssier, Dirk van der Marel, & Michel C. Milinkovitch

Additional file 5: Angular dependence of the multilayer reflectivity

The angle of propagation of light in a multilayer is a parameter that modifies the different optical paths and thus shifts the main reflection peak. We have measured the angular dependence of the reflectivity for the purely structural peak of the *P. grandis* #1 sample (cf. Figure 3a in the main text). The experimental setup (described in [1]) uses the Ocean Optics spectrometer coupled to the variable angle spectroscopic Woollam ellipsomter. Using the fibered spectrometer does not allow measuring in the UV, but it is fast (this is essential because exposure of skin to air quickly modifies the structural color component, see movie in Additional file 4). Results of the measurements in the range of 20 to 50 degrees are shown in Figure S5. A blue shift of about 15 nm can be seen, much smaller than that predicted by the model (inset). This weak angular dependence probably arises from the fact that: *(i)* the orientation of the iridophores varies substantially even in blue skin (Figure S5b), *(ii)* the reflectivity is measured over a 2 mm^2 area covering several skin scales, each having a curvature that already averages over a wide angular range.

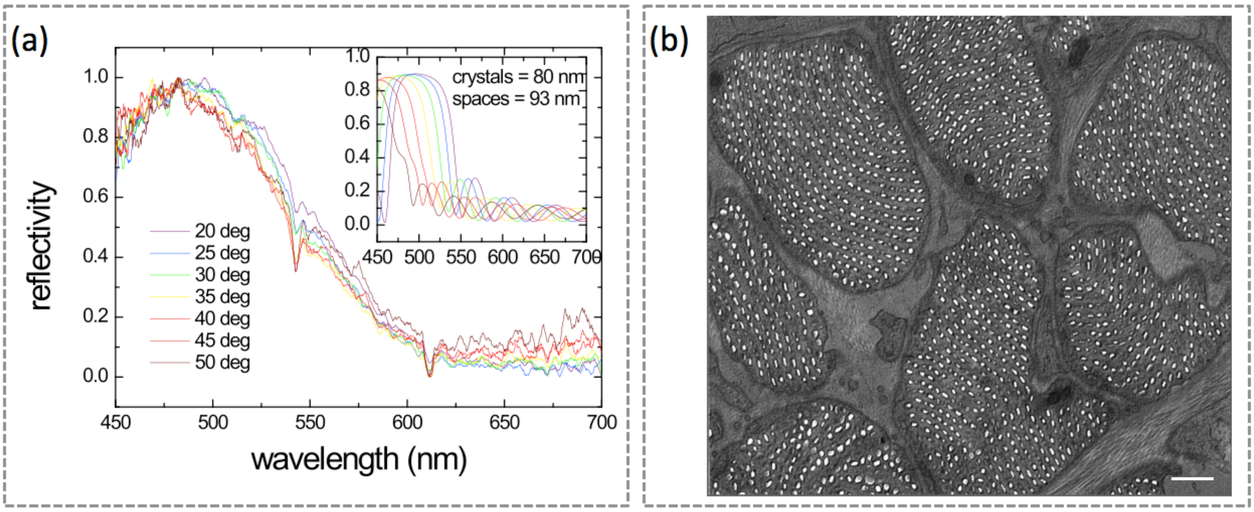

Figure S5. (a) Angular dependence of the reflectivity measured on *P. grandis* #1 between 20 and 50 degrees. Inset shows angular dependence, of light with polarization at 45°, of the multilayer model for crystal size of 80 nm and spacing of 93 nm. (b) TEM image showing variable orientation of iridophores relative to skin surface (*i.e.*, even though the nanocrystals are well organized in parallel layers within each cell, the orientation of different cells can vary substantially). Bar = 1 micron.

References:

[1] Dufresne ER, Noh H, Saranathan V, Mochrie SGJ, Cao H, Prum RO: **Self-assembly of amorphous biophotonic nanostructures by phase separation.** *Soft Matter* 2009, **5**:1792–1795.
